# Dr. Morris O. Pitcher

**Published:** 1917-02

**Authors:** 


**Dr. Morris O. Pitcher,** (data as graduation lacking in Polk, not listed in State Directory) died at his home in Sardinia, Jan. 16.

